# Genomic Advancements in Assessing Growth Performance, Meat Quality, and Carcass Characteristics of Goats in Sub-Saharan Africa: A Systematic Review

**DOI:** 10.3390/ijms26052323

**Published:** 2025-03-05

**Authors:** Keabetswe T. Ncube, Khathutshelo A. Nephawe, Takalani J. Mpofu, Nare J. Monareng, Mbongeni M. Mofokeng, Bohani Mtileni

**Affiliations:** Department of Animal Sciences, Tshwane University of Technology, Pretoria 0002, South Africanarejessicamonareng@gmail.com (N.J.M.); mbongeni2896@gmail.com (M.M.M.);

**Keywords:** genomics, growth performance, meat and carcass quality traits, goat breeds, genomic selection

## Abstract

Goats play a vital role in global livestock systems, particularly in developing regions, where they contribute significantly to meat production and smallholder livelihoods. Indigenous goats in sub-Saharan Africa are essential to low-input farming systems, valued for their adaptability to harsh environments and their provision of meat, milk, and income. However, genomic research on these goats remains limited despite their importance. Recent advancements in genomic technologies, such as next-generation sequencing (NGS), genome-wide association (GWAS) studies, and single nucleotide polymorphism (SNP) mapping, have identified key genes like *MSTN*, *IGF1*, and *CAST*. These genes influence muscle growth, fat deposition, and meat tenderness, which are critical for improving growth performance, carcass characteristics, and meat quality. Genomic selection offers a promising avenue for enhancing economically valuable traits, such as faster growth rates and adaptability to challenging climates. This review highlights the potential of integrating genomic tools with traditional breeding practices to optimise goat production systems, enhance meat quality, and improve economic outcomes for farmers. It also underscores the need for further research to fully characterise the genetic diversity of indigenous goat breeds in sub-Saharan Africa. Addressing these knowledge gaps could significantly contribute to the region’s food security and sustainable farming practices.

## 1. Introduction

Goats are a cornerstone of livestock production in sub-Saharan Africa, where they contribute significantly to household income and food security, especially in arid and resource-constrained environments. Their adaptability to diverse climatic conditions, ability to thrive on marginal lands, and minimal resource requirements make them ideal for commercial and subsistence farming systems [1,2]. Valued for their meat (chevon), milk, fibre, and skins, goats provide versatile livelihood options for smallholder farmers in resource-constrained environments. Across many communities in Africa and Asia, goats hold significant economic and cultural value, symbolising wealth, social security, and status [3,4].

Goats’ capacity to adapt to tough weather circumstances and the growing demand for chevon and milk is driving the goat population’s notable expansion in both the eastern and western areas of sub-Saharan Africa (SSA) [5,6]. According to recent projections, there are over 372 million goats in SSA overall, with a significant rise in the number of goats raised for milk production [5]. This expansion is critical for improving food security and reducing poverty. In Kenya, the Galla goat population has been maintained as a pure breed in the government breeding station under artificial selection for many generations, known for its potential for meat, milk production, and better survival under harsh tropical conditions [7]. China leads global production, accounting for more than 25% of the world’s goats, followed by India, Pakistan, and Nigeria [1]. In South Africa, approximately 7.8 million goats are reared, with 78% raised by communal farmers, underlining their importance for rural socio-economic stability [8].

The global distribution of goats highlights significant distinctions between commercial and communal farming systems. Commercial goat farming is primarily practised in developed countries, where modern breeding strategies, genetic selection, and improved nutrition maximise production efficiency. For example, Australia is among the world’s largest importers of goat meat (Table 1), focusing on breeds like the Boer goat, known for its rapid growth and superior carcass quality [9].

Although there are notable regional differences, the present market demand for goat meat in sub-Saharan Africa is marked by a growing interest. Countries such as Nigeria, Ethiopia, and Sudan rank among the largest goat producers (FAO, 2022), while in Tanzania, goat meat is second in significance after cattle, and consumers favour the hind leg since it is the meatiest piece [10]. Goat meat is acknowledged for its nutritional value in South Africa, especially in KwaZulu-Natal, although it is still less popular than other meats like chicken and beef, due to cultural customs and restricted supply [11]. Indigenous goats demonstrate resilience to disease, drought, and poor-quality feed, making them vital in areas where other livestock species may not thrive [12].

**Figure 1:**
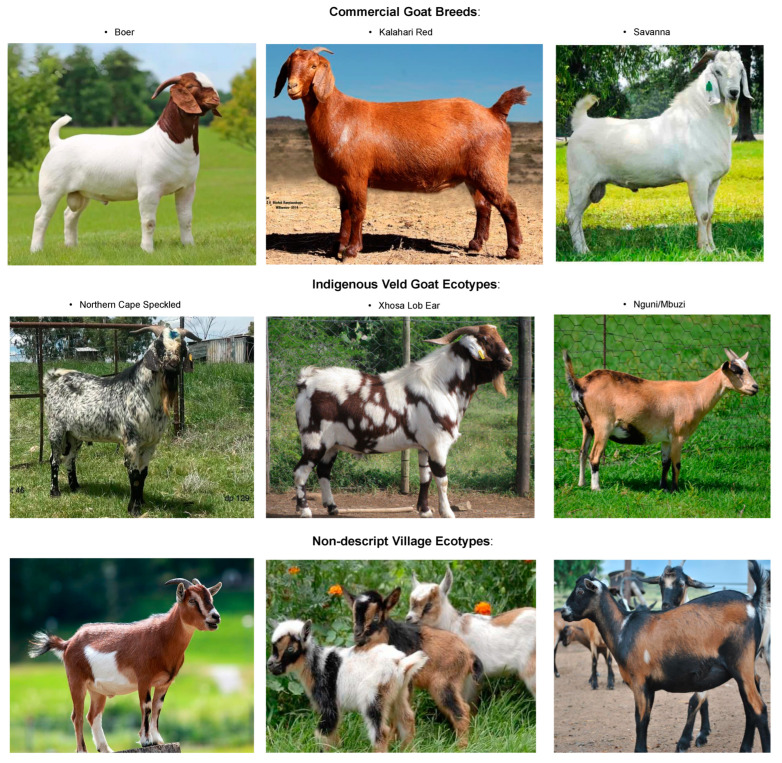
South African goat populations and their different production systems.

In South Africa, a variety of established goat breeds and structured production systems exist, including the South African Boer, Kalahari Red, and Savannah, primarily used for meat production [2,13,14] under the commercial production system (Figure 1). Indigenous Veld Goats include the Northern Cape Speckled, Xhosa Lob Ear, and Nguni/Mbuzi, among others [8,15]. Non-descript goat populations raised under the extensive systems represent valuable genetic resources that could enhance food security and livelihoods in marginalised communities. Improved management practices, such as controlled feeding and veterinary care, can further enhance the growth performance of these village goats by mitigating environmental challenges [8].

Meat and carcass quality are essential traits in livestock production [16], making genomic studies of goat populations crucial for identifying candidate genes related to growth and meat quality. Recent genomic research has highlighted key genes, such as *myostatin* (*MSTN*) and *protein kinase AMP-activated non-catalytic subunit gamma 3* (*PRKAG3*), associated with growth and carcass traits [17,18]. Mutations in the *growth hormone 1* (*GH1*) gene have been linked to phenotypic variation in growth, presenting opportunities for selective breeding [19].

**Figure 2:**
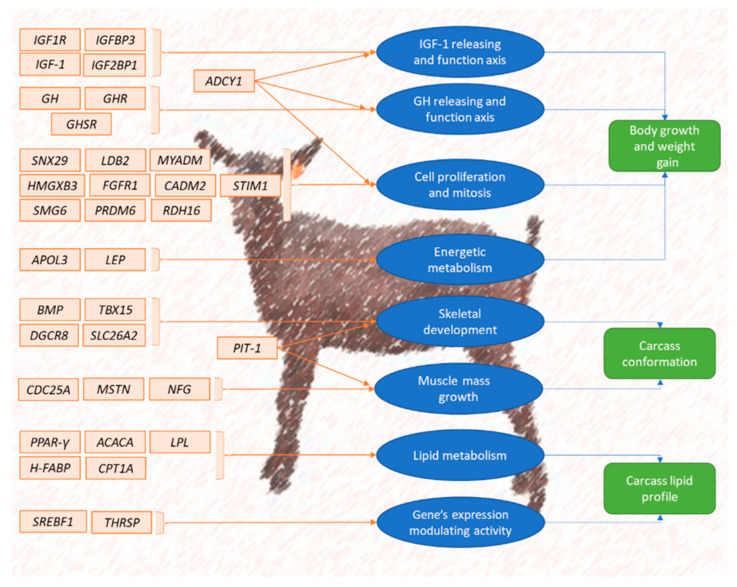
Summary scheme of caprine candidate genes’ influence on meat production [22].

Various studies have employed genome-wide association (GWAS) studies and high-throughput sequencing to uncover genetic variations related to growth rates, carcass composition, and meat tenderness [2,18,20]. Research on South African Boer goats has identified significant genes like *growth differentiation factor 8 myostatin* (*GDF8*), *calpastatin* (*CAST*), and *leptin* (*LEP*), linked to muscle growth, fat deposition, and meat quality [2] (Figure 2). These genetic markers correlate with superior carcass traits, including increased muscle mass and improved tenderness. Integrating such genetic data into selective breeding programmes promises to enhance the productivity and sustainability of indigenous goat breeds, contributing to South Africa’s agricultural economy [21].

Figure 2 illustrates the interplay between various genetic pathways and goat meat production traits, such as body growth, carcass conformation, and lipid profiles. Genes associated with growth hormone and IGF-1 pathways (*IGF1R*, *ADCY1*, *GH*, *IGF-1*) primarily influence body growth and weight gain. Those regulating cell proliferation, energetic metabolism, and muscle growth (*PRDM6*, *SMG6*, *FGR1*, *LDB2*) have more specific effects on tissue development, influencing the yield and quality of meat. Genes involved in lipid metabolism shape the carcass lipid profile, contributing to meat quality in terms of intramuscular fat and overall texture. This integrative genetic approach is essential for understanding and improving caprine meat production traits through selective breeding. This approach combines genomic tools, such as GWAS and next-generation sequencing, with traditional breeding practices to identify key genetic markers associated with traits like muscle growth, fat deposition, and carcass quality. This enables targeted selection of genetically superior animals, accelerates genetic gains, and provides insights into gene-environment interactions, thereby optimising breeding programmes tailored to specific production systems.

The genetic improvement of goats through selective breeding has long been recognised as vital for enhancing productivity, particularly regarding growth performance and meat quality [2]. Traditional breeding methods have often been inadequate in low-input systems, where environmental factors can outweigh genetic potential. However, advancements in genomic technologies provide transformative opportunities to accelerate genetic gains by identifying and selecting key traits [23]. Genomic technologies, such as QTL mapping, genome-wide association (GWAS) studies, gene editing, and genomic selection (GS), have revolutionised livestock breeding by enabling the identification of genes associated with economically important traits [24]. In goats, these technologies have facilitated the discovery of genetic factors influencing traits such as growth performance, muscle development, and meat tenderness, which are crucial for productivity in both commercial and communal systems [2]. Additionally, the genetic characterisation of indigenous sub-Saharan African goats—such as eastern Africa’s Maasai and Mubende, southern Africa’s Ndebele and Pafuri, and western Africa’s Djallonke and West African Dwarf—using microsatellite markers has revealed significant variability within and between populations [25]. These, along with next-generation sequencing (NGS) technologies, facilitate the development of reference genomes and genome-wide association studies (GWASs), enabling high-resolution mapping of the goat genome, including the improved goat reference assembly, providing insights into traits like body weight, carcass yield, and meat quality [26]. Integrating genomic data with phenotypic records enhances the accuracy of estimated breeding values (EBVs) by enabling the calculation of genomic EBVs (gEBVs), which can predict an animal’s genetic potential at an early age, thereby improving breeding efficiency [23].

This systematic review aims to comprehensively examine the current state of genomic research in goats, focusing on growth performance, carcass characteristics, and meat quality traits. It specifically explores the application of advanced genomic tools, including next-generation sequencing (NGS) technologies, genome-wide association studies (GWAS), and single nucleotide polymorphism (SNP) mapping, which has revolutionised livestock genetics. This review underscores the transformative potential of integrating genomic research into breeding strategies to address key challenges in goat farming, such as low productivity, inconsistent meat quality, and limited adaptability to harsh environments. By synthesising findings from recent studies, it aims to provide a detailed understanding of genes influencing economically important traits in goats. Furthermore, this review highlights how genomic tools can complement traditional breeding practices to accelerate genetic gains, improve selection accuracy, and enhance livestock productivity in resource-constrained systems, such as those prevalent in sub-Saharan Africa. It also identifies critical research gaps and proposes future directions to unlock the full potential of genomic advancements in goat breeding.

## 2. Methods

This systematic review followed the Preferred Reporting Items for Systematic Reviews and Meta-Analyses (PRISMA) guidelines to collect and evaluate pertinent literature about genomic technologies employed in goat research, with a particular emphasis on Next Generation Sequencing (NGS) and non-NGS tools. Studies were identified through a comprehensive search of databases such as Web of Science, PubMed, Scopus, and Google Scholar, using the keywords “genomics”, “metagenomics”, “sub-Saharan African goats”, “goats”, “meat quality traits”, “growth performance”, “carcass characteristics/traits”, “NGS technologies”, “SNP arrays”, “RNA-Seq”, “whole-genome sequencing”, “GWAS in goats”, “genetic diversity in goats”, and “growth traits”. Inclusion criteria were peer-reviewed articles published in English between 2000 and 2024, focusing on applying genomic tools to indigenous goat populations or challenges specific to sub-Saharan Africa, such as environmental adaptability, disease resistance, and productivity in low-input systems goats. Exclusion criteria were non-peer-reviewed studies, articles not focusing on goats and those that did not employ genomic techniques.

## 3. Results and Discussion

**Figure 3:**
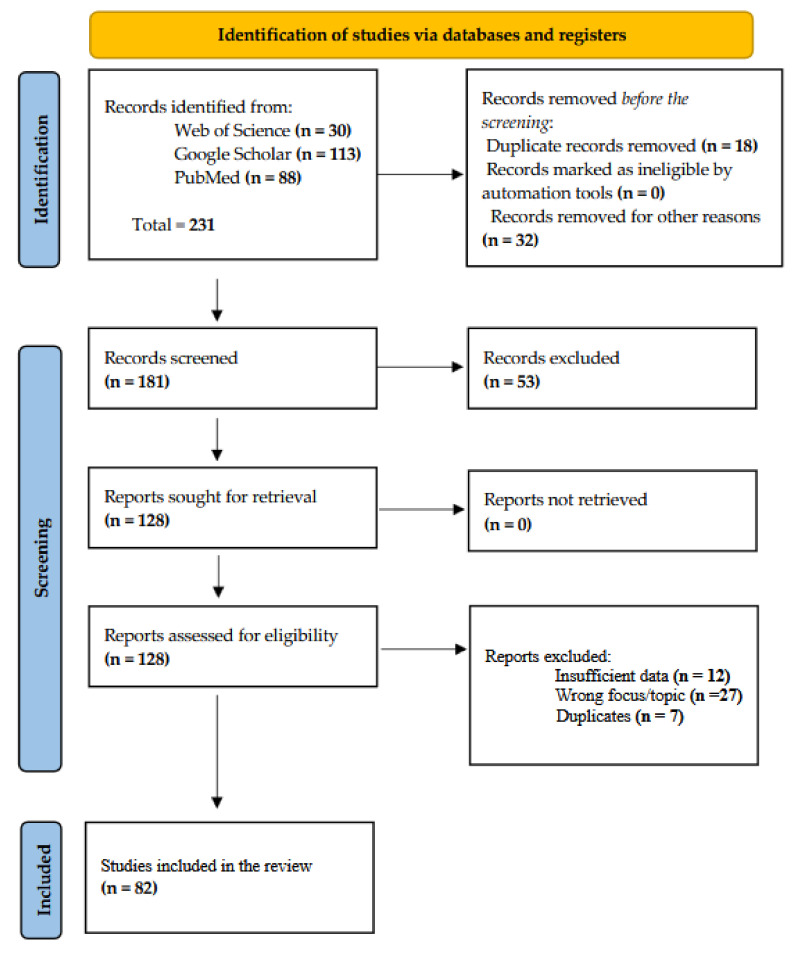
PRISMA flow diagram adapted from Page et al. (2021) [27].

Studies focusing on the application of genomic technologies in goats, especially those examining characteristics like growth rates, growth performance, meat, meat quality and carcass characteristics, were required to meet the exclusion criteria. Studies that did not specifically centre on goats or were not subjected to peer review were excluded based on specific criteria. Eighty-two studies were included in the final review after 231 publications were screened for relevancy (Figure 3).

These investigations included more recent NGS-based techniques like RNA-Seq and whole-genome sequencing in addition to more established non-NGS techniques like SNP arrays and microsatellite markers. The review employs a comparative analytical methodology, classifying and evaluating each genomic technique according to its uses, benefits, drawbacks, and contributions to the field of goat genomics. Tables summarising genomic advancements were revised to emphasise traits and genetic tools relevant to sub-Saharan African goat populations. A comparative analysis between indigenous and commercial breeds within the region was included to highlight the key findings.

### 3.1. Growth Performance in Goat Populations

Growth is a crucial quantitative trait that significantly influences mature body weight and overall productivity within goat production systems (Table 2) [28]). Among various performance traits, growth rate, particularly pre- and post-weaning growth rate, serves as a key indicator of adaptation to environmental and management conditions [29]. Growth rate is typically measured as an increase in body weight and is affected by multifactorial influences, including nutrition, genetics, and management practices [6]. Growth performance encompasses various metrics, with average daily gain (ADG) being a critical parameter. This measurement reflects the rate of weight gain over time, and it is highly influenced by the quality of feed, making it a key determinant of productivity.

In goat production systems, disparities in management practices—such as the distinction between communal and commercial farming—play a crucial role in growth outcomes. Communal goats forage for food with limited nutritional support [8,38], while goats in large-scale commercial farms benefit from adequate feed, veterinary care, and intensive management. Although nutrition and management practices are important, genetics remains a fundamental driver of growth performance. A deeper investigation into the genetic factors influencing growth performance, particularly under communal farming conditions, is necessary to enhance goat productivity. Examining the growth profiles and carcass quality traits of goats can provide a better understanding of the growth potential of goats, particularly local populations.

Growth performance in goats is influenced by a combination of polygenic traits—phenotypic characteristics controlled by multiple genes with small additive effects—and specific genetic determinants, such as major genes like *IGF-1* and *MSTN*, which have a larger impact on traits like growth rate, body composition, and overall development. Numerous genetic factors, including breed-specific growth performance, genetic markers, heritability, and quantitative trait loci (QTL), influence the genetic variation underlying growth traits in goats, thereby affecting their overall growth potential. Genomic selection (GS) utilises genetic information to predict the genetic potential for economically important traits, such as growth rate and feed efficiency, thereby improving the accuracy of selection for these traits. For instance, South African Boer goats, renowned for their rapid growth and superior feed conversion efficiency, demonstrate the impact of targeted genetic selection, with weaning weights typically ranging from 25 to 30 kg and an average daily gain (ADG) of 200–300 grams under optimal conditions [1]. In comparison, the Kalahari Red goat exhibits commendable adaptability to harsh climates, achieving mature weights of around 48.99 kg for does and 61.75 kg for bucks [32]. Studies comparing traditional selection methods with genomic selection have shown significant improvements in prediction accuracy and genetic progress, highlighting genomic selection as a powerful tool for optimising growth performance in goat breeding programmes.

Indigenous breeds, such as the Xhosa lob-ear and Nguni/Mbuzi goats, while generally slower-growing, are well-adapted to local conditions and contribute significantly to the livelihoods of smallholder farmers, showcasing weaning weights of 15 to 25 kg and an ADG often below 200 grams [8]. Comparative studies have highlighted significant variability in growth performance among different breeds. Research by Snyman (2014) [37], Pophiwa et al. (2017) [30], and Ncube et al. (2022a) [2] demonstrates that Boer goats consistently outperform village ecotypes in terms of live weight and carcass quality. This superior growth is attributed to their genetic makeup, favouring muscle development and higher growth rates. In contrast, non-descript village ecotypes, though adapted to challenging environmental conditions, typically exhibit slower growth rates and lower carcass yields, which can be ascribed to both genetic predispositions and environmental factors. These comparisons underscore the importance of selecting appropriate breeds based on specific production goals and environmental contexts, illustrating the trade-offs between rapid growth and adaptability in meat goat populations globally. Moreover, heritability estimates for growth traits in goats often range from 0.25 to 0.4, indicating that these traits are moderately influenced by genetic factors. This suggests that selective breeding, when combined with accurate phenotypic and genomic data, can lead to genetic improvements over generations, optimising growth traits in goat populations [39]. This genetic variability can be effectively exploited in breeding programs, particularly through the use of estimated breeding values (EBVs), to select animals with optimal growth characteristics, especially in crossbreeding initiatives involving high-performing meat breeds Boer and village goats. Several quantitative trait loci (QTLs) have been identified in goats associated with growth traits, including those linked to body weight, feed intake, and overall growth efficiency. Polymorphisms in genes like *growth hormone* (*GH*), *insulin-like growth factor-1* (*IGF-1*), and *myostatin* (*MSTN*) have been shown to influence growth rates and muscle development [8]. Studies in South Africa have highlighted the potential for marker-assisted selection (MAS) to enhance growth performance in Boer goats. Genetic diversity is a critical factor for sustaining growth performance. Inbreeding, common in small populations or closed breeding systems, can lead to inbreeding depression, negatively affecting growth rates. To counteract this, South African goat breeders should maintain diverse breeding lines, especially in indigenous breeds that are often subjected to communal farming practices with limited genetic management. The expression of genetic growth potential is also highly dependent on environmental factors. In resource-limited communal farming systems, even goats with superior genetic potential may underperform due to insufficient nutrition and inadequate veterinary care [40]. Identifying genetic lines that perform well under local environmental conditions is crucial for improving growth performance in these systems.

Despite the research that has been carried out on growth performance, there is still a gap in understanding the genetic factors influencing growth at various developmental stages of goats. Investigating growth profiles and characterising genetic determinants at different stages of growth (birth, weaning, post-weaning, and maturity) are essential for optimising breeding strategies. Growth profiling using methods such as body measurement indices [13,28,41] and body weight measurements are valuable tools for assessing growth potential. These methods could be particularly useful in communal farming systems where formal record-keeping is lacking.

With the increasing establishment of community-based breeding programs in Africa, there is a need to integrate genetic profiling with traditional body measurements to improve growth performance in communal farming systems. Chest girth measurements, for example, have been employed in rural Ethiopia to estimate live weight in small ruminants where weighing scales are unavailable [34]. Such practical, low-cost methods could be beneficial for monitoring and enhancing growth performance in South African goat populations, particularly in resource-limited settings.

### 3.2. Carcass Characteristics in Goat Populations

Carcass characteristics are fundamental to the profitability and sustainability of the goat meat industry, significantly influencing both producer and consumer preferences. Understanding the genetic and phenotypic variations in carcass traits between commercial and non-descript village goats is crucial for improving production efficiency and enhancing meat quality. Genomic selection offers a promising avenue for advancing these objectives by enabling breeders to identify and propagate animals with desirable carcass traits at the molecular level. Studies [2,30] have highlighted the importance of genomic selection for carcass characteristics in South African goat populations, with a focus on key traits such as carcass weight, dressing percentage, muscle composition, and conformation [2,30].

Carcass weight and dressing percentage are pivotal determinants of a goat’s market value as they directly impact both the quantity and quality of meat produced. Dressing percentage refers to the ratio of the dressed carcass weight to the live animal weight, expressed as a percentage [42]. This trait is influenced by genetics, nutrition, and management practices, with commercial goats typically exhibiting higher dressing percentages than non-descript village goats due to selective breeding and improved feeding regimens. In the South African context, breeds such as Boer, Kalahari Red, and Savanna goats dominate the commercial sector, with genetic selection for optimal carcass weight being a key driver of profitability. Genomic selection facilitates the identification of genetic markers associated with higher dressing percentages, allowing for more efficient and targeted breeding programmes. The importance of dressing percentage in South African goats has been emphasised, noting that commercial breeds consistently outperform village goats in this regard due to advancements in selective breeding and feed optimisation [2]. The study underscores the potential for genomic selection to further enhance these traits, particularly in the commercial sector, where carcass weight is a critical factor in determining market value.

Carcass composition—specifically the proportions of muscle, fat, and bone—plays a significant role in determining meat quality and consumer preferences. With an increasing demand for leaner, low-fat meats, the muscle-to-fat ratio has emerged as a key factor in carcass quality [43]. Commercial goat breeds, such as the Boer and Kalahari Red, generally exhibit a higher muscle-to-fat ratio compared to village goats, largely due to selective breeding for feed conversion efficiency and muscle development. Genomic technologies, including next-generation sequencing, have enabled the identification of genes involved in muscle growth, such as those regulating myogenesis (muscle fibre formation).

Genomics research has explored the genetic basis of carcass composition in South African goats, revealing key genetic variations that influence muscle and fat deposition [2]. The previous study highlights the potential of genomic selection to enhance the muscle-to-fat ratio in commercial breeds, thereby increasing meat yield and improving carcass quality. However, challenges remain in applying these genomic tools to non-descript village goats, which exhibit a wider range of genetic and phenotypic variability [8,13,14,31,43]. Carcass conformation refers to the shape and structural attributes of the carcass, with a particular focus on the development of specific body regions such as the hindquarters, loin, and rib cage—areas that produce the most desirable cuts of meat [44]. In South Africa, commercial breeds such as the Boer goat have been selectively bred for superior conformation, resulting in well-developed hindquarters and a greater loin eye area, which are indicative of higher meat yield. Genomic selection provides an opportunity to further refine these traits by identifying and propagating animals with favourable genetic profiles for carcass conformation [2,23]. Ncube et al. (2022a) [2] noted that South African commercial goats consistently outperform village goats in terms of carcass conformation, largely due to the emphasis placed on selective breeding for specific traits. The above-mentioned study also highlighted the potential for genomic selection to enhance carcass conformation, particularly in the hindquarters and loin, which are critical regions for high-value meat cuts. While genomic selection presents a promising avenue for improving carcass characteristics, several challenges persist, particularly in the context of South African goat populations. One of the primary challenges is the genetic diversity present in non-descript village goats. Genetic diversity hinders the consistency of genomic markers across populations, reducing predictive accuracy and requiring multi-population models for effective selection.

Furthermore, environmental factors such as nutrition, management practices, and climatic conditions can obscure the expression of alleles or genes associated with genetically favourable traits, further complicating selection efforts. Studies have identified the lack of comprehensive genomic data for South African goats as a significant barrier to the widespread implementation of genomic selection [2,15,40,45]. This gap in data limits the ability of breeders to develop robust genomic tools tailored to local conditions, particularly for village goats, which may not exhibit the same phenotypic responses as commercial breeds under similar genetic selection pressures. Therefore, while genomic selection offers significant potential for enhancing carcass characteristics in goats, particularly in commercial breeds, challenges related to genetic diversity, environmental variability, and data availability must be addressed to fully realise these benefits. Previous studies provide a foundation for future research and breeding programmes aimed at improving the carcass traits of both commercial and village goats.

### 3.3. Meat Quality Traits in Goat Populations

Meat tenderness is a critical determinant of consumer preferences, influencing repeat purchases and the willingness to pay premium prices [46]. Factors such as sarcomere length, protein denaturation, intramuscular fat content, connective tissue structure, and myofibrillar integrity contribute significantly to meat tenderness [46]. In South African goats, there is a marked difference in tenderness between commercial breeds, such as Boer and Kalahari Red, and non-descript village goats. Commercial goats typically produce more tender meat, a trait often attributed to selective breeding for muscle structure and enhanced post-mortem proteolysis. The identification of genetic markers linked to tenderness-related genes, particularly those encoding proteolytic enzymes like calpains and cathepsins, could facilitate the genomic selection of goats for improved meat tenderness. Studies focusing on South African goats have underscored the genetic influences on tenderness, pointing to specific gene regions that influence post-mortem muscle degradation and proteolysis [2,47]. These studies have highlighted the potential for using genomic selection to optimise tenderness traits in both commercial and village goat populations. By leveraging genomic selection to estimate genome-enhanced breeding values (GEBVs) for tenderness, breeders can make more informed selection decisions, ultimately improving overall meat quality for consumers.

Meat colour is another critical quality trait that strongly influences consumer acceptance, as it is often associated with freshness and perceived quality. The primary protein responsible for meat colour is myoglobin, while cytochromes and haemoglobin also contribute, albeit to a lesser extent [46]. A key factor affecting meat colour is the final pH of the meat, with significant colour changes occurring when the meat’s pH deviates from its optimal range [47]. Post-mortem pH is influenced by both genetic factors and pre-slaughter stress [46], making it an essential consideration in meat quality control. Genetic influences on meat colour and pH regulation stem from genes that control glycolytic enzyme activity and muscle energy metabolism. Genomic selection for these traits has the potential to ensure more consistent meat quality in goats. Commercial goat breeds, which are typically raised under more controlled conditions, often produce meat with more desirable colour and pH levels compared to village goats. The genetic underpinnings of these differences have been associated with specific genes regulating muscle energy metabolism and stress responses, which, in turn, influence meat pH and colour. The ability to select favourable genetic profiles in these traits could improve consumer satisfaction and marketability of goat meat.

Genomic selection offers a transformative approach to improving meat quality traits such as tenderness, colour, and pH in goats. By leveraging advances in next-generation sequencing technologies, researchers have been able to identify key genetic loci associated with these traits, facilitating more targeted breeding programmes. Genomic selection has the potential to bridge the quality gap between commercial and village goats, improving the competitiveness of the latter in the marketplace. It has been observed that certain genes associated with proteolytic enzymes (such as calpains) are linked to tenderness, while genes influencing glycolysis and muscle energy metabolism play a critical role in regulating meat pH and colour. The identification of these genetic markers opens new opportunities for breeders to enhance meat quality traits in goats, particularly in commercial breeds, where consumer demand for premium meat quality is high.

Genomic selection for meat quality traits presents a significant opportunity to enhance the goat industry, particularly by improving tenderness, colour, and pH. While challenges exist, especially concerning the genetic diversity of village goats, genomic selection using genome-enhanced breeding values provides a promising pathway for breeders to improve meat quality through more precise selection.

### 3.4. Applications of Genomics Tools in Goat Production

Goat genomics has advanced significantly with the introduction of both next-generation sequencing (NGS) and non-NGS tools, which have transformed the study of genetic diversity, trait inheritance, and disease resistance. NGS technologies provide comprehensive genetic data that can be rapidly analysed, offering deep insights into the genetic makeup of goat populations. Non-NGS tools, including SNP arrays and microsatellite markers, while more traditional, remain integral to population genetics and selective breeding programmes. It is, therefore, important to explore the primary genomic tools used in evaluating meat, carcass, and growth performance traits in goat populations, outlining their applications, benefits, and limitations.

The application of genomic tools has facilitated a more detailed understanding of the genetic mechanisms underlying economically significant traits in goats. Several genes have been associated with key traits such as milk, fibre, and meat production, disease resistance, reproduction, and growth [22,38,48]. Notably, genes like *leptin* and caprine *myostatin* are linked to carcass quality and growth performance (Table 3). For instance, leptin has been shown to influence weight gain, making it a valuable marker for enhancing meat production [49]. Similarly, caprine *myostatin* is associated with improved meat tenderness [50], highlighting its potential use in selective breeding for desirable meat characteristics.

Table 3 highlights the roles of key genes such as *MSTN*, *IGF1*, and *CAST* in influencing growth performance, meat quality, and carcass traits in livestock, identified through genomic tools like GWAS, QTL mapping, and RNA sequencing, which assess genetic variation based on a predefined set of SNPs. These genes play vital roles in regulating economically important traits. For instance, *myostatin* (*MSTN*) inhibits muscle growth, with mutations linked to increased muscle mass. In Charolais and Limousine sires, a specific mutation in the *MSTN* gene has been linked to carcass weight, fat content, and body conformation. It has also been associated with enhanced muscular development in the hindquarters and inner thigh and increased thigh width in both breeds [57,58]. Research has shown that sequence analysis of the *MSTN* gene identified six SNPs, with four shared across breeds and two monomorphic in Ossimi, Rahmani, and Najdi but polymorphic in Barki. Notably, c.18 G>T and c.241 T>C were significantly associated with birth weight and average daily weight gain, respectively [59]. On the other hand, *MSTN* mutations in goats moderately increase muscle growth while maintaining adaptability to diverse environments, making it a critical target for selective breeding in resource-constrained systems [60].

The effects of growth hormone are primarily mediated by the *IGF1* gene, which stimulates body growth and has growth-promoting effects in almost every cell of the body, such as skeletal muscle, bone, etc., [38]. A study on indigenous goats raised under different production systems demonstrated the high expression of *IGF1* in intensively raised goats vs. those raised under extensive systems. This further shows the association of growth performance with *IGF1* and the positive impact of production systems on the realisation of genetic potential in village goats [38]. However, the genetic impact of *IGF1* in goats appears less pronounced compared to other livestock, likely due to breed-specific selection pressures and gene-environment interactions that merit further research [61]. The *insulin-like growth factor 1* (*IGF1*) gene has been recognised as a key biological candidate for traits related to growth, body composition, metabolism, and skeletal development. Additionally, it serves as a positional candidate gene for growth and fat deposition in chickens [62]. It plays a crucial role in the development and growth of various tissues, including muscle and bone [63]. A study conducted on broiler chickens in the Czech Republic revealed that certain *IGF1* genotypes exhibited the highest average body weight at 42 days in both chicken lines. These genotypes were also associated with increased average abdominal fat weight, breast muscle weight (with or without skin), and thigh muscle weight (with or without skin), as well as improved slaughter value and slaughter percentage in both lines [64]. Furthermore, the *calpastatin* (*CAST*) gene regulates proteolytic enzymes, significantly influencing meat tenderness [2]. In cattle, *CAST* polymorphisms are strongly associated with improved tenderness, juiciness, and overall meat quality, making it a key genetic marker in breeding programmes. In goats, CAST variations also impact tenderness and carcass characteristics; however, the limited number of studies highlights the need for additional research to validate its utility in goat breeding. In South African goat populations, high-throughput next-generation sequencing (NGS) has revealed polymorphic variations in genes such as the *growth hormone 1* gene, shedding light on genetic diversity across breeds and production systems [65]. Genomic tools like SNP arrays are critical for identifying single base-pair modifications, which explain genetic differences among animals and contribute to performance traits [66]. These advanced technologies offer valuable insights into the genetic potential of South African goats, particularly non-descript village goats, and hold promise for enhancing growth and meat production through targeted breeding strategies. These findings underscore the consistency of these genes’ influence across species while highlighting the importance of tailoring genomic selection strategies to specific production systems. Leveraging cross-species insights and genomic tools such as GWAS, RNA-Seq, QTL mapping, and SNP arrays can help optimise goat breeding programmes and improve economically important traits.

#### 3.4.1. Genome-Wide Association Studies (GWAS)

Genome-wide association studies (GWASs) have become an essential tool in animal genomics, particularly for identifying common genetic variants linked to traits by genotyping populations with known phenotypic information. These studies reveal genotype-phenotype correlations, especially in complex traits like growth and reproduction, improving breeding selection strategies [54,67]. When integrated with Quantitative Trait Loci (QTL) mapping, GWAS enhance the understanding of trait variation [68]. Advances in next-generation sequencing (NGS) have further refined GWAS, detecting rare variants and providing deeper insight into the genetic architecture of complex traits in goats, such as body weight and growth, with significant contributors being genes like *SIX6* and *TRPS1* [54,69].

In the context of goats, GWASs have been pivotal in uncovering the genetic basis of economically important traits such as growth performance, meat quality, and carcass characteristics. For instance, research has identified the most significant genes for body weight (*CRADD*, *HMGA2*, *MSRB3*, *MAX*, *HACL1*, and *RAB15*) that regulate growth processes, body sizes, fat deposition, and average daily gains (ADG) in Karachai goats [34]. In South African goat populations, GWASs have been employed to study growth traits, carcass characteristics, and genetic diversity. Waineina et al. (2022) [7] found that candidate genes associated with adaptation in tropical environments include *MST1R*, *HYAL1/2/3*, and *SFRP1*, which regulate biological processes like cellular response to heat stress and disease resistance. For instance, the Illumina SNP50K chip identified higher carcass weights in Boer goats compared to indigenous breeds, with traits influenced by sex, age, and breed [2]. It also revealed differences in bone formation and body measurements between communal indigenous goats [43]. This technology has also revealed SNPs that are common to body weight and localised within a window of 200 kb that were found from the *CRADD*, *HMGA2*, *MSRB3*, *FUT8*, *MAX*, and *RAB15* structural genes [35]. Moreover, studies have shown the SNP50K chip’s usefulness in analysing population structures, such as the identification of the feral Tankwa population as genetically distinct from other South African goats [31]. Population structure studies shed light on the understanding of goat populations and their variations; this can aid in understanding how various populations differ in growth performance and meat quality traits. The *HMGA2* gene is widely recognised as a key candidate involved in prenatal and early postnatal development, while the *MSRB3* gene has been suggested as a potential candidate influencing growth performance [34]. Some SNPs linked to growth, body metabolism, and adaptive traits were found to segregate among goat populations in different geographic environments in South Africa [14].

This technology has also proven its usefulness in studying the meat quality traits of goats. An investigation into meat productivity and the chemical composition of muscle tissue in 8-month-old Karachai goats with different genotypes, based on identified SNPs, revealed that SNP rs268269710 holds the greatest potential for use in selective breeding for meat traits [35]. A study in Tashi goats identified several SNPs and candidate genes that may influence body conformation traits [55]. In addition, two key regions (chr.10:25988403-26102739 and chr.11:88216493-89250659), along with several candidate genes, such as *FNTB*, *CHURC1*, and *RNF144*, highlighted in this study, merit further investigation and could be applied in the molecular breeding of meat goats.

Carcass traits, such as dressing percentage, carcass weight, and muscle-to-fat ratio, are critical for the profitability of goat production systems. A recent GWAS study in South African indigenous goat breeds has uncovered SNPs linked to these traits, identifying genes such as *LEPR* and *MC4R*, which are involved in fat deposition and body composition regulation [2]. These findings offer genetic markers that can be incorporated into breeding programmes to enhance carcass yield and quality.

Despite these advancements, there remain gaps in understanding the genetic factors influencing growth performance, carcass characteristics, and meat quality in South African goats, especially relative to their potential in the chevon industry. Further research is required to fully exploit GWAS and improve these economically important traits in goat breeding programmes. The SNP genotyping array tools, such as the Goat_IGGC_65K_v2 BeadChip, can be employed to identify single nucleotide polymorphisms (SNPs) associated with these traits. Genomic studies should prioritise the identification of genomic regions linked to key production traits, including growth performance, carcass characteristics, and reproductive efficiency. Advanced GWAS tools, such as mixed linear models (MLMs) and Bayesian approaches, can enhance the detection of quantitative trait loci (QTLs) while accounting for population structure and kinship.

Additionally, the development of population-specific reference genomes and SNP panels tailored to indigenous goat populations is essential for improving the resolution and accuracy of GWAS findings. Software tools like PLINK, GEMMA, and GCTA can aid in the identification and annotation of genetic variants associated with these traits. Furthermore, despite the economic and ecological importance of indigenous goat populations, research on their genomic diversity and potential remains limited in sub-Saharan Africa [25].

#### 3.4.2. Next-Generation Sequencing (NGS)

Next-generation sequencing (NGS) technologies have become essential in modern genomics, particularly for high-resolution genetic analysis in species such as goats. The main NGS methods used in goat research include RNA sequencing (RNA-Seq), whole-genome sequencing (WGS), and shotgun metagenomics. These NGS techniques enable the simultaneous analysis of millions of DNA sequences, greatly enhancing genetic research [70]. Compared to traditional methods like microarrays, NGS offers improved accuracy and versatility [71]. It allows for exploring transcriptomes without prior gene knowledge, yielding insights into gene variations and alternative splicing. Notably, platforms such as the Illumina HiSeq 2500 and NovaSeq 6000 can generate vast amounts of sequence data within short timeframes. It has transformed the identification of genetic markers associated with important livestock traits; for example, WGS has facilitated the discovery of various genomic variants critical for production efficiency. RNA-Seq, on the other hand, has been crucial for analysing gene expression related to meat quality and carcass traits, and studies by Saccà et al. (2019) [56] and Devapriya et al. (2021) [70] identified differentially expressed genes associated with muscle fibre composition and tenderness, underscoring the role of NGS in improving meat quality through genetic insights.

In population genomics, NGS has enabled the assessment of genetic diversity and the detection of selection signatures in goat populations. Studies, such as those by Dzomba et al. (2018) [14], have shown how NGS can elucidate genetic differentiation in South African goat populations, revealing loci tied to body size and metabolic efficiency. This highlights NGS’s potential to inform breeding strategies that enhance growth performance and adaptability.

#### 3.4.3. Whole-Genome Sequencing (WGS)

Whole-genome sequencing (WGS) has become a transformative tool in animal genetics, allowing researchers to investigate an organism’s entire genetic makeup with high precision. WGS offers insights into coding and non-coding regions of the genome, facilitating the identification of genetic variants that underlie important economic traits in livestock. This employs the high sequence coverage required to generate sufficient data, often necessitating over 6.3 million reads per sample [71,72].

Whole genome sequencing has played a crucial role in advancing goat genomic studies, such as a study on the South African Tankwa feral goat that revealed several genetic variants associated with key cellular pathways, defence mechanisms, and immunity [72]. These findings highlight the potential of WGS in identifying single nucleotide polymorphisms (SNPs) that influence growth performance, disease resistance, and adaptation processes in goats [72]. Moreover, WGS has been instrumental in identifying selection signatures related to traits such as coat colour, reproduction, and high-altitude adaptation [52,73]. Notably, WGS has also contributed to the discovery of candidate genes associated with economically significant traits. A study on sheep revealed genes that influence the number of thoracic vertebrae, a trait that directly affects carcass length and meat yield [74]. These findings have significant implications for improving carcass traits in goats, as they provide insights into genomic regions that could be targeted in selective breeding.

The first draft genome of a goat, achieved through WGS, was that of the Yunnan black goat, with a genome size of approximately 2.66 Gb [75]. This reference genome paved the way for subsequent genomic studies, enabling the identification of structural variants such as copy number variations (CNVs) and SNPs. The use of WGS in the development of reference genomes has been invaluable in exploring genetic diversity and evolutionary relationships among different goat breeds.

Fatty acids are required for daily normal metabolism and are an important trait contributing to meat quality [56]. In another goat study, the *CAB39L* gene was identified through WGS, which has also been instrumental in uncovering genetic determinants of meat quality, a complex trait influenced by genetics, diet, and environmental factors [56]. Genomic analyses have revealed variations in the *FABP4* gene, which plays a role in intramuscular fat deposition, affecting meat tenderness and flavour [52]. Furthermore, the *CAST* gene, which encodes *calpastatin*, a protease inhibitor, has been linked to meat tenderness by regulating muscle degradation post-slaughter [52]. These insights are invaluable for improving meat quality through selective breeding, allowing producers to cater to consumer preferences for tenderness and flavour.

#### 3.4.4. RNA-Sequencing

RNA-Seq is a high throughput sequencing method for gene expression profiling widely used for mapping and quantifying transcriptomes and analysing gene expressions in various tissues [76], providing more accurate levels of transcripts used to measure transcriptome composition and discover new exons or genes [73]. RNA-Seq studies have shown that gene knockout has a positive impact on muscle growth and development in goats [73]. RNA-Seq technology has also proven its usefulness in the gene expression profiling of the intramuscular muscle in Nellore cattle, where several genes that were associated with lipid metabolism and fatty acid composition were identified [77]. Transcriptome analysis studies were able to associate expressed genes to pathways that play a role in cell stimulations and neutral growth in chickens, as well as phenotypes in slow and fast-growing chickens [78]. These, among many other studies, demonstrate the potential for RNA-Seq as a tool to be used for gene expression profiling, as there are associations that can be linked to growth traits. The transcriptome of any tissue is affected by factors like breed and physiological and environmental conditions, which makes RNA-Seq experimental designs challenging as these conditions need to be controlled to reduce noise and obtain accurate associations. Because of these requirements, RNA-Seq studies work very well for commercial breeds or experimental populations that have uniform genetics, constant production systems, good and uniform management systems, etc. The design of RNA-Seq experiments for extensively raised populations is challenged by genetic variations and inconsistent production and management systems. In an uncontrolled environment, a high number of differentially expressed genes may be observed.

Despite the challenges of setting up a transcriptomic study, especially in the village populations, Ncube et al. (2022b) [38] investigated the gene expression profiles related to growth performance and carcass traits in South African goats. This study observed that genes such as *GH1* and *IGF1*, which are central to growth regulation, were upregulated in goats raised under intensive production systems, indicating better growth performance due to favourable conditions. Notably, the intensively raised village goats (VTIs) exhibited significantly higher live weights compared to extensively raised goats (VTEs), with higher expression of genes like *POU1F1*, *CHKB*, and *BAG4* associated with growth and muscle development. The findings highlight the genetic basis for differences in growth performance and carcass quality between breeds and production systems, with the intensively raised goats showing superior growth metrics.

Though there has been advancement in the genomic studies of South African goat populations, transcriptomics is one method that has not been explored to address some of the research questions for this sector. The majority of the goats in South Africa are raised in communal farms with no structured management and breeding systems [65]; moreover, when coupled with the high genetic variation in village goats, this may present challenges in setting up gene expression profiling studies.

#### 3.4.5. Metagenomics

Metagenomics, a vital next-generation sequencing (NGS) tool, enables the comprehensive study of microbial communities in diverse samples, including those that cannot be cultured through conventional methods [79]. This approach allows researchers to investigate microbial diversity and functionality, offering insights into the role of microbes in various biological processes that impact host organisms. Amplicon sequencing, especially targeting the 16S rRNA gene in bacteria and archaea, facilitates the efficient profiling of microbial taxa by amplifying hypervariable regions, enabling the identification of microbial signatures within complex environments [80,81].

In livestock research, shotgun sequencing further enhances metagenomic studies by enabling the analysis of all genes present in a sample, thus providing a more detailed view of community structure, genetic diversity, and functional capacities [69,82]. In goats, this method is particularly valuable for exploring the gut microbiome, which plays a crucial role in digestion, nutrient absorption, and overall health. Metagenomics allows researchers to examine the interactions between host genetics, diet, and the microbiome, shedding light on how these factors influence growth performance, meat quality, and carcass traits [69]. Despite the recognised potential of metagenomics, there is a notable scarcity of studies. This gap limits our understanding of how microbial communities influence key production traits such as growth rates, carcass composition, and meat quality, particularly in these under-researched populations. The gut microbiome significantly influences the efficiency of nutrient utilisation in goats, which directly impacts growth performance. Metagenomic analyses have identified microbial taxa and functional genes associated with enhanced feed conversion efficiency, fibre digestion, and volatile fatty acid production—key factors in ruminant growth [83]. By examining the diversity and functional potential of these microbial communities, researchers can identify microbial markers associated with superior growth performance. This knowledge can be applied to develop microbial-based interventions, such as targeted probiotics or prebiotics, aimed at optimising nutrient utilisation and promoting growth in goats.

Metagenomics also offers valuable insights into carcass traits, such as fat distribution, muscle-to-bone ratio, and overall body composition. Microbial communities play a critical role in protein and lipid metabolism, processes that influence muscle growth and fat deposition [84]. By identifying microbial pathways involved in nitrogen utilisation and protein synthesis, metagenomics can reveal microbial contributions to greater muscle mass and improved carcass yield. Moreover, the microbiome’s interaction with the endocrine system, particularly in modulating growth-related hormones, may impact fat and muscle distribution, further influencing carcass characteristics.

Regarding meat quality, metagenomics has demonstrated that gut microbiota affects fat deposition, muscle development, and overall health, which, in turn, influence traits such as tenderness, flavour, and marbling. The microbiota plays a key role in lipid metabolism, which determines intramuscular fat content and marbling—critical factors for meat quality [85]. However, microbiome studies, particularly those that investigate the relation between gut microbiota and growth, carcass characteristics, and meat quality traits have not yet been performed in sub-Saharan Africa, demonstrating a further research gap. Understanding the relationship between microbial diversity and lipid biosynthesis pathways could help in selecting microbial communities that promote favourable meat quality attributes. Such insights could lead to the development of microbial interventions aimed at improving meat characteristics like tenderness and flavour through the targeted modulation of host metabolic processes.

## 4. Conclusions and Recommendations

Advancements in goat genomics have significantly improved our understanding of well-established meat goat breeds. However, a critical and pressing gap remains in the genetic characterisation of non-descript village goats, which constitute a substantial portion of goat populations, particularly in sub-Saharan Africa. These indigenous goats, shaped by centuries of natural selection, represent a reservoir of unique genetic diversity, enabling them to thrive under challenging environmental conditions. Yet, their genetic potential for growth performance, carcass characteristics, and meat quality traits remain underexplored. This oversight continues to hinder the ability of resource-constrained smallholder farmers to fully benefit from these locally adapted populations. Profiling their growth at various developmental stages is not merely a scientific endeavour but an urgent necessity to unlock their full genetic potential, enabling the identification of traits valuable for improving meat yield and quality.

From a South African perspective, the lack of comprehensive data on the carcass characteristics of village goat populations presents significant limitations for the design of effective breeding and management strategies. This shortfall perpetuates the inefficiency of local goat production systems, which are crucial for food security and rural livelihoods. Effective breeding strategies must prioritise not only productivity but also adaptability, resilience, and sustainability, traits often overlooked in traditional improvement programmes. Genomics tools, including next-generation sequencing (NGS), genome-wide association studies (GWAS), and QTLs, offer the transformative potential to address these issues by unravelling the complex genetic networks that underpin growth performance, carcass traits, and meat quality. However, deploying these tools in sub-Saharan Africa, especially in smallholder systems, requires critical and immediate attention to the accessibility, affordability, and practicality of these technologies in resource-limited environments.

While genomic tools have revolutionised livestock breeding globally, their adoption in sub-Saharan Africa has been impeded by several barriers. The high costs associated with advanced genomic technologies, the technical expertise required for their application, and the limited infrastructure in rural areas are significant challenges. These hurdles disproportionately affect smallholder farmers, who form the backbone of goat production in the region. To bridge this gap, future research must go beyond genetic characterisation to actively address these systemic limitations. Practical solutions such as the development of low-cost genomic platforms, simplified data analysis pipelines and targeted capacity-building initiatives are essential to make these tools accessible to smallholder farmers. Moreover, the integration of genomic data into breeding programmes must be context-specific, considering the socio-economic realities, resource constraints, and traditional practices of rural farming communities. Establishing community-based breeding programmes that merge genomic selection with traditional selection criteria has the potential to not only enhance growth performance and meat quality but also preserve the unique genetic diversity of indigenous goat populations. Such programmes should prioritise traits that are economically valuable and relevant to smallholder farmers, such as disease resistance, environmental adaptability, and improved carcass quality, ensuring that the benefits of genomic advancements are equitably distributed.

The application of genomic tools has already identified key genes, such as *MSTN*, *IGF1*, and *CAST*, which regulate economically important traits, including muscle growth, fat deposition, and meat tenderness. Leveraging this knowledge in breeding strategies tailored for resource-limited settings could yield significant gains in productivity and profitability for smallholder farmers. However, the success of these initiatives hinges on multi-stakeholder collaboration. Research institutions must work together with government agencies, rural leadership, and local communities to develop policies, frameworks, and funding mechanisms that support the adoption of genomic technologies. Governments and policymakers have a critical role in subsidising genomic research, developing rural infrastructure, and facilitating knowledge transfer to farmers. Equally important is fostering a culture of participatory research, where smallholder farmers are actively involved in the identification of breeding objectives and the implementation of genomic tools. Building trust and ensuring that farmers understand the tangible benefits of genomic selection will be key to driving adoption. Training programmes for extension officers, rural educators, and farmers themselves will be vital in equipping communities with the skills and knowledge to integrate genomic information into their breeding decisions. Furthermore, partnerships with international organisations and private sector stakeholders can help mobilise resources and expertise to accelerate the deployment of these technologies.

In conclusion, the conservation and genetic characterisation of indigenous village goat populations in sub-Saharan Africa is not only a scientific imperative but a socio-economic necessity. Expanding data collection on traits such as growth performance, carcass characteristics, meat quality, and environmental adaptability will empower breeding programmes to bridge the performance gap between commercial and village goats. By integrating genomic tools into these programmes, we can unlock the genetic potential of indigenous goats, improving productivity, resilience, and sustainability in goat farming systems. These efforts will require a concerted and coordinated approach, combining investment in genomic research, infrastructure development, and community engagement. Collaborative partnerships between research institutions, governments, rural leadership, and farming communities are essential to ensure that the benefits of genomic advancements reach those who need them most. By addressing the accessibility and practicality of these technologies, we can transform goat farming in sub-Saharan Africa, not only enhancing food security and livelihoods but also preserving the invaluable genetic resources of indigenous goat populations for future generations.

## Figures and Tables

**Table 1:** Top goat importers in the world from 2011 to 2021 [9].

Country	Average Value (Tonnes)	% of the Total Volume
Australia	26,818.61	45
Ethiopia	13,797.09	23
Kenya	4143.68	7
France	2459.98	4
Pakistan	2416.82	4
China	2212.84	4
Spain	2056.20	3
New Zealand	1183.23	2
Jordan	884.40	1
Other countries	3424.81	7

**Table 2:** Various goat breeds and their growth performance, meat quality, and carcass-related traits.

Goat Breed	Growth Performance	Meat Quality	Carcass Characteristics	References
Boer	Rapid growth rates (200–300 g/day), superior feed conversion efficiency	High tenderness, low-fat content, favourable intramuscular fat content	Higher dressing percentage (~47–50%), increased muscle mass	[2,30]
Kalahari	Fast growth rates, adaptable to harsh climates	Lean meat, lower fat content, good tenderness	Moderate carcass yield, favourable muscle-to-fat ratio	[31,32]
Savanna	Average growth rate	High meat tenderness and palatability	High dressing percentage and conformation	[33]
Xhosa Lob Ear	Average growth rates (~150-200g/day), adult weight is 32 kg for bucks and29 kg for does	Acceptable meat tenderness, lower intramuscular fat content	Low dressing percentage, lower muscle-to-bone ratio	[8,34]
Nguni/Mbuzi	Slower growth rates (~120 g/day) with an average adult weight of 40 kg in bucks and 34 kg in does	Lower tenderness and intramuscular fat content	Low carcass yield	[2,14,35]
Karachai	Moderate growth rates (~150–200 g/day)	Lean meat, good fat distribution	Higher dressing percentage, good muscle-to-fat ratio	[36,37]

**Table 3:** Genes associated with growth performance, meat quality, carcass characteristics and the genomic tools used for their identification.

Gene	Role	Associated Trait (s)	Genomic Tool (s)	References
*MSTN*, *MYF5*	Inhibits muscle growth; mutations lead to increased muscle mass. Controls muscle differentiation and growth	Muscle growth, carcass yield	GWAS, QTL mapping, WGS	[2,14,29,51]
*IGF1*	Regulates growth and development of muscle tissue	Growth rate, muscle development	RNA-Seq, Whole Genome Sequencing	[14,35]
*CAST*, *CAPN1*	Inhibits proteolytic enzymes; affects muscle fibre degradation post-mortem. Encodes calpain, affecting muscle fibre breakdown and tenderness post-slaughter	Meat tenderness	QTL mapping, GWAS, SNP Array	[2,30]
*LEP*	Regulates appetite and energy balance, impacting fat storage and feed efficiency	Fat deposition, growth efficiency	SNP Array, GWAS	[2,49]
*GDF8*	Promotes muscle growth and repair	Carcass weight, muscle mass	QTL mapping, GWAS	[2,30]
*PRKAG3*	Influences energy metabolism in muscle, impacting fat deposition and muscle composition	Muscle-to-fat ratio, carcass yield	GWAS, SNP Array	[2,17]
*MC4R*	Regulates feed intake and energy balance, influencing lean muscle growth	Lean growth, feed efficiency	GWAS, QTL mapping	[3,43]
*FABP4*	Binds and transports fatty acids, influencing intramuscular fat and marbling	Fat deposition, marbling	RNA-Seq, Whole Genome Sequencing	[38,52]
*CHKB*	Involved in phospholipid synthesis, impacting muscle formation and carcass quality	Muscle development	QTL mapping, WGS	[53,54]
*RNF144*	Involved in muscle and tissue development, influencing carcass structure	Carcass conformation	GWAS	[35,55]
*FST*	Inhibits muscle regulatory factors, influencing growth rate and carcass quality	Growth performance	RNA-Seq, GWAS	[2,14]
*HMGA2*	Regulates cellular growth processes, associated with body size and carcass traits	Body size, growth rate	SNP Array, GWAS	[34,54]
*CAB39L*	Associated with lipid metabolism, affecting meat quality and flavour	Fatty acid composition	Whole Genome Sequencing	[52,56]
*MAX*	Regulates cell proliferation and differentiation, impacting growth rate and body structure	Growth regulation, body composition	GWAS, SNP Array	[34,35]

## Data Availability

There were no new data analysed for this review.
